# Tissue expression of Squamous Cellular Carcinoma Antigen (SCCA) is inversely correlated to tumor size in HCC

**DOI:** 10.1186/1476-4598-8-29

**Published:** 2009-05-27

**Authors:** Paolo Trerotoli, Emilia Fransvea, Umberto Angelotti, Giovanni Antonaci, Luigi Lupo, Antonio Mazzocca, Anita Mangia, Salvatore Antonaci, Gianluigi Giannelli

**Affiliations:** 1 Department of Biomedical Science and Human Oncology, Section of Medical Statistics, University of Bari Medical School, Bari, Italy; 2 Department of Internal Medicine, Immunology and Infectious Diseases, Section of Internal Medicine, University of Bari Medical School, Bari, Italy; 3 Department of Emergency and Organ Transplantation, University of Bari Medical School, Bari, Italy; 4 Vanderbilt University Medical Center Department of Pathology, Nashville, USA; 5 Clinical Experimental Oncology Laboratory, National Cancer Institute Bari, Italy

## Abstract

**Background:**

This study aimed to investigate squamous cellular carcinoma antigen (SCCA) in serum and in tumoral and paired peritumoral tissues. We studied 27 patients with liver cirrhosis (LC) and 55 with HCC: 20 with a single nodule < 3 cm (s-HCC) and 35 with a single nodule > 3 cm or multifocal (l-HCC).

**Methods:**

Serum SCCA was measured by the ELISA kit, and in frozen tissues by immunohistochemistry, quantified with appropriate imaging analysis software and expressed in square microns. Continuous variables are reported as means and 95% confidence intervals. Comparisons between independent groups were performed with a generalized linear model and Tukey grouping. Pearson's correlation coefficients were determined to evaluate relations between markers. Qualitative variables were summarized as count and percentage. Statistical significance was set at p-value < 0.05.

**Results:**

Serum SCCA values in LC patients were 0.41 (0.31–0.55) ng/ml and statistically different from both HCC groups: 1.6 (1.0–2.6) ng/ml in s-HCC, 2.2 (1.28–2.74) ng/ml in l-HCC. SCCA in hepatic tissue was 263.8 (176.6–394.01) μm^2 ^in LC patients, statistically different from values in s-HCC: 1163.2 (863.6–1566.8) μm^2 ^and l-HCC: 625.8 (534.5–732.6). All pairwise comparisons between groups yielded statistically significant differences. Tumoral SCCA resulted linearly related with nodule size, showing a statistically significant inverse relation between the two variables (b = -0.099, p = 0.024).

**Conclusion:**

There was no statistically significant correlation between tissue and serum levels of SCCA. The significantly stronger expression of SCCA in smaller compared to larger HCC could be important for early HCC detection. However, the increased expression in peritumoral tissue could affect the significance of serological detection.

## Background

Early recognition of the onset of hepatocellular carcinoma (HCC) would help to select more effective therapies for patients, leading to a better prognosis and life span. For this reason surveillance programs were strongly recommended at a Consensus Conference held in Barcelona [1]. Alphafetoprotein (AFP), the only marker commonly used in clinical practise, displays poor sensitivity and a high specificity only for values higher than 400 IU/ml. However, because AFP concentrations are directly correlated with tumor size, the reliability of such a marker appears inadequate for early recognition of HCC [2]. This has prompted a high number of studies conducted to validate different new biomarkers, but very little has yet been reported about biomarkers helping to achieve an early detection of HCC [3]. All the proposed biomarkers failed to discriminate between liver cirrhosis (LC) and HCC in a satisfactory manner, in terms of diagnostic accuracy, reproducibility of the results, or technical issues related to the biomarker detection method [4]. For this reason, the simultaneous use of different tests seems to offer a promising approach that warrants further investigation [5].

Squamous cellular carcinoma antigen (SCCA), is a member of the high molecular weight family of serine protease inhibitors named serpins [6]. Two highly homologous isoforms have been reported to be expressed in HCC tissues at protein and translational levels [7]. SCCA has also been reported to be overexpressed in tumoral compared to paired peritumoral tissue of HCC, suggesting a role as a potential marker for histological detection of HCC [8]. Recently, SCCA has been investigated in regenerative and dysplastic nodules of HCC tissue. Interestingly, results show that SCCA was poorly expressed in regenerative tissue but strongly increased in dysplastic nodules, suggesting a role as a potential marker for early detection of HCC [9].

Aim of this study is to investigate the tissue expression of SCCA in patients with different characteristics of HCC, namely small and large or multifocal nodules.

## Results

SCCA was quantified in matching sera and tissues of 82 patients, 27 LC and 55 HCC. In these latter patients tissue expression of SCCA was investigated in neoplastic and paired peritumoral tissue. Patients were stratified according to nodule size. The mean (95%CI) size of the HCC lesion was 4.07 ± 2.08 cm; 36.4% (20/55) patients had a single nodule smaller or equal to 3 cm (s-HCC) and 63.6% (35/55) a nodule larger than 3 cm or multifocal (l-HCC). A statistically significant difference (p = 0.01) was observed in the percentage of Child-Pugh stage A among s-HCC (55%), l-HCC (82%) and LC (88%). The most frequent etiology was HCV alone or with HBV, with no significant difference among groups (p = 0.108): 65% (15/20) in s-HCC, 71.4% (25/35) l-HCC and 92.6% (25/27) for LC patients (Table 1).

**Table 1:** Characteristics of the patients

		Single nodule ≤ 3 cm	Single nodule > 3 cm	Total HCC	LC
Sex	M	16	28	44	17
	F	4	7	11	10
					
Age		65 (10.15)	65 (10. 2)	65 (10.1)	
					
Size		2..4 (0.5)	5 (2)	4 (2.1)	
					
CLIP	0	7	15	22	
	1	10	17	27	
	2	3	3	6	
					27
					
CHILD	A	11	29	40	24
	B	9	6	15	3
					
Etiology	Alcohol or Other	1	2	3	1
					
	HBV	3	8	11	1
	HBV+Alcohol	1		1	
	Total HBV	4	8	12	1
	HCV alone	13	23	36	24
	HCV+HBV	1	2	3	
	HCV+Alcohol or other	1		1	1
	Total HCV	15	25	40	25

**Figure 1:**
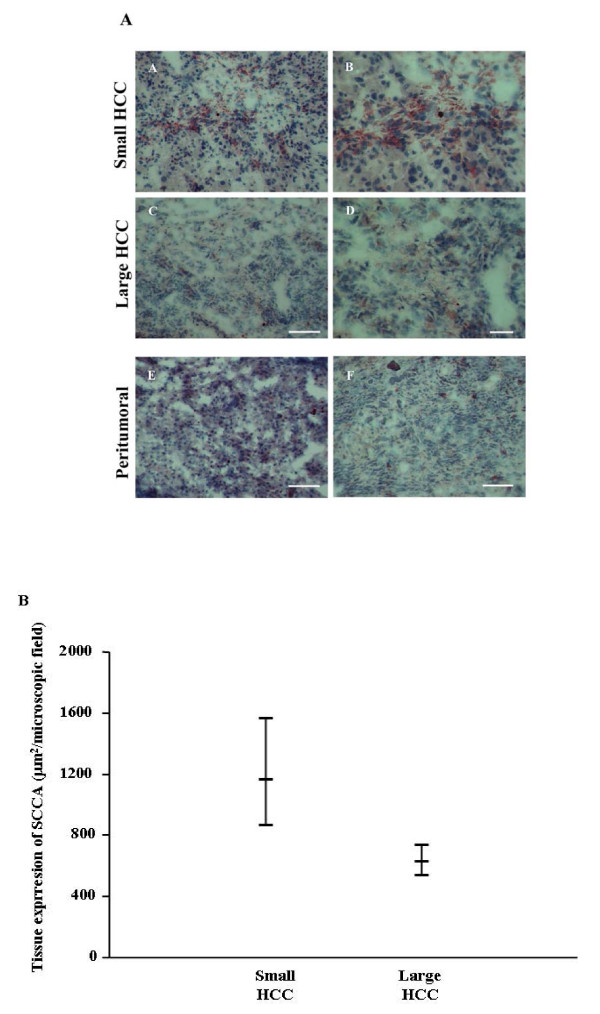
**Tissue expression of SCCA**. In Figure 1A, immunohistochemistry of SCCA in small and large HCC at low (A, C) and high (B, D) magnification. Paired peritumoral tissue at low magnification of small (E) and large (F) HCC. In A, C, E and F scale bar = 100 μm, in B and D scale bar = 50 μm. In Figure 1B, 95% Confidence intervals of neoplastic tissue SCCA in the different HCC patients groups.

**Figure 2:**
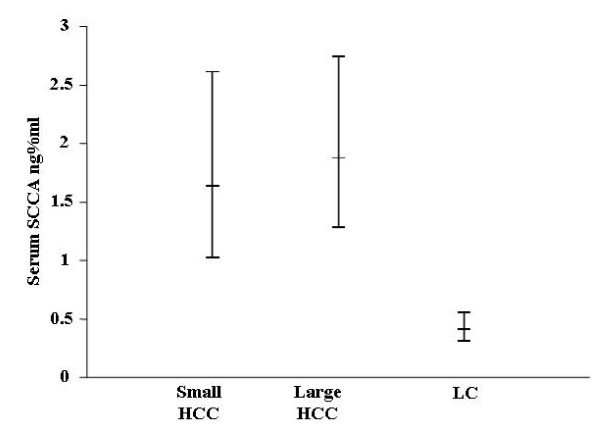
**95% Confidence intervals of non neoplastic peritumoral tissue SCCA in the different HCC patients groups**.

SCCA was detected by immunohistochemistry in all the patients, although with some differences, in both tumoral and peritumoral tissues of small and large HCC (Figure 1A). In particular, the mean (95% CI) expression in neoplastic tissue was 1163.2 (863.6–1566.8) μm^2 ^in s-HCC and 625.8 (534.5–732.6) μm^2 ^in l-HCC. There was a statistically significant difference among the groups (F = 17.45, p = 0.002) and Tukey grouping showed a significant difference (p < 0.05) between s-HCC vs l-HCC (Figure 1B). To further investigate the tissue expression of SCCA, paired peritumoral tissues were analyzed as well as LC samples used as proper control. The mean (95% CI) of SCCA tissue expression was 263.8 (176.6–394.01) μm^2 ^in s-HCC peritumoral tissue, 345.49 (263,98 – 452,16) μm^2 ^in l-HCC and 232.63 (163.3–331.38) μm^2 ^in LC, Figure 2. The model did not result statistically significant (F = 1.84, p = 0.16). In conclusion, SCCA was more strongly expressed in the neoplastic tissue of smaller compared to larger HCC. In addition, it is noteworthy that the ratio between tumoral and peritumoral SCCA shows a trend ranging from 4.4 in s-HCC to 1.8 of l-HCC.

**Figure 3:**
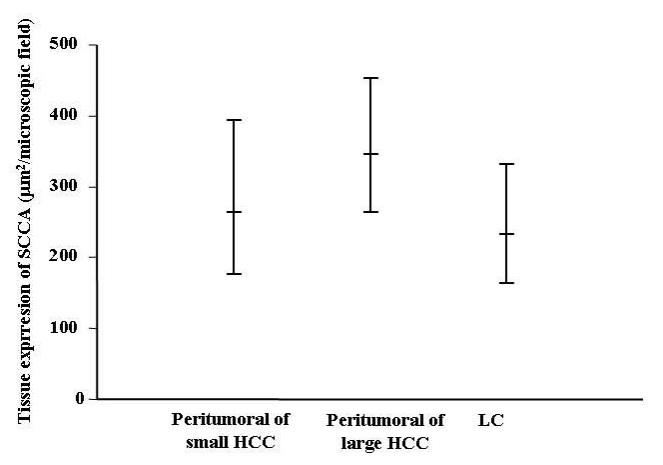
**95% Confidence intervals of serum SCCA in the different HCC patients groups**.

In the same patients, we measured the serum concentrations of SCCA. As reported in Figure 3, concentrations were 1.6 (1.02–2.6) ng/ml in s-HCC, 2.2 (1.28–2.74) ng/ml in l-HCC, 0.41 (0.31–0.55) ng/ml in LC patients (Figure 3). The model resulted statistically significant (F = 20.81, p < 0.0001), and Tukey grouping displayed a significant difference (p < 0.05) between each HCC group and LC. In conclusion, serum SCCA levels were similar in the different HCC groups, but statistically different in the LC group.

The underlying liver disease does not seem to affect SCCA tissue expression levels among patients with different Child-Pugh stages (p = 0.5) as shown by the generalized linear model, while the only significant factor remains the diagnosis of HCC as compared to LC (p = 0.049).

The regression model with dependent peritumoral SCCA shows that in the subgroup of single nodule HCC, nodule size was not statistically significant as a means of predicting peritumoral SCCA (b = 0.064; F = 1.55; p = 0.219), whereas it was a statistically significant predictor of tumoral SCCA (b = -0.099; F = 5.47; p = 0.024; R^2 ^= 0.117).

Linear regression between serum SCCA and nodule size did not show a statistically significant relation between these two variables (b = 0.023; F = 0.14; p = 0.71; R^2 ^= 0.003).

In conclusion, tumoral SCCA depends on nodule size, and there was an inverse trend between nodule size and higher SCCA values. Peritumoral and serum SCCA, instead, do not show any relation with tumor size.

The area under the ROC curve for serum SCCA was 0.897 (CI95% 0.81–0.953), with a suggested cut-off value of 1.1 ng/ml, showing 72.7% sensitivity, 100% specificity and a 72.7%Youden index. Assessing only s-HCC and LC patients, an analogous accuracy of serum SCCA was obtained: AUC 0.873 (CI95% 0.743–0.952), cut-off 1.1 ng/ml, with 70% sensitivity, 100% specificity and 70% Youden index.

However, in HCC no statistically significant correlation was observed between SCCA levels present in the tumoral and/or peritumoral tissue and in the serum (Table 2). Pearson's correlation coefficient resulted statistically significant only to evaluate relations between serum levels vs cirrhotic liver tissue expression of SCCA in LC patients: 0.39 (p = 0.04). In other words, the higher the tissue values the higher the serum values, but only in LC patients.

**Table 2:** Pearson correlation coefficient for evaluation of relation between markers in HCC and LC patients

		Peritumoral SCCA	Tumoral SCCA
s-HCC	Serum SCCA	0.01	-0.08

	Tumoral SCCA	0.27	

			

l-HCC	Serum SCCA	0.05	-0.13

	Tumoral SCCA	0.014	

			

LC	Serum SCCA	0.39 (p = 0.04)	

**Figure 4:**
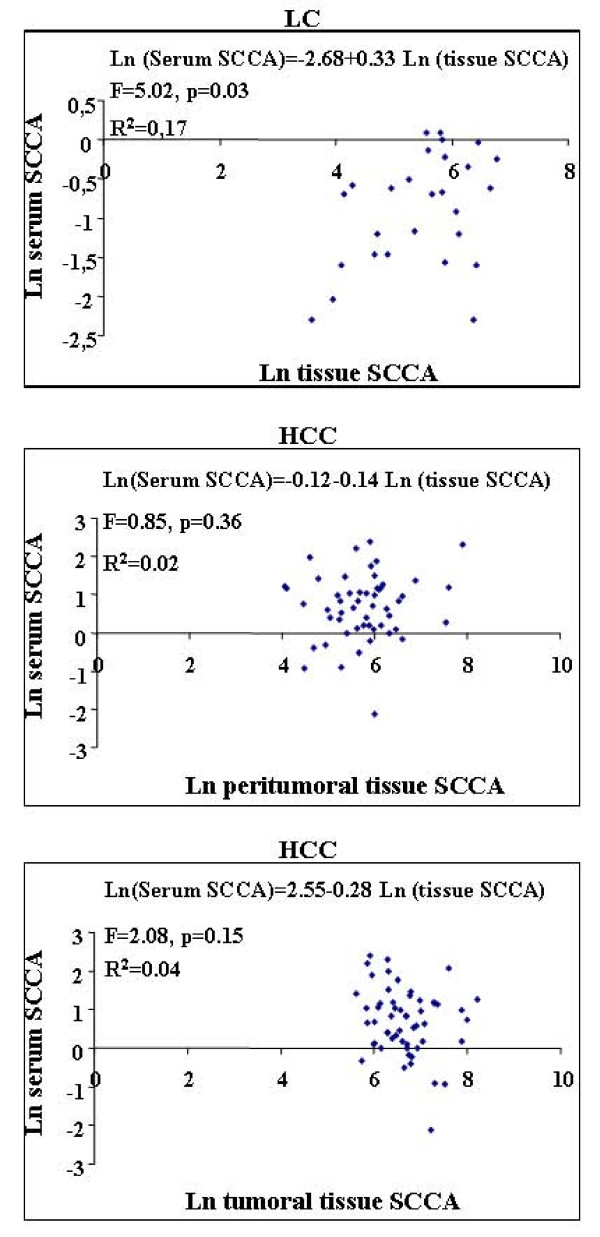
**Scatter plot of serum SCCA in tumoral and peritumoral tissue in HCC patients, and tissue SCCA in LC patients**. Ln = natural logarithm.

Finally, serum SCCA values could be expressed as a function of tumoral or peritumoral values of this marker, so a regression analysis was performed; results are shown in Figure 4. The models evaluated did not result statistically significant except in LC patients (F = 5.02, p = 0.034). In this group the slope resulted 0.33 (p = 0.034), suggesting increased SCCA serum values with stronger tissue expression. In HCC patients, neither SCCA tumoral nor peritumoral tissue expression resulted predictive of the serum levels, and this finding was consistent in both groups of patients. Furthermore, in smaller nodules we calculated that 711 μm^2 ^of SCCA antigen were necessary to measure one unit in the serum, while in larger tumors only 268 μm^2 ^were enough.

## Discussion

Lately, the investigation of new biomarkers for HCC diagnosis has aroused great interest because they could make it possible to select the most effective therapy for individual patients. From this viewpoint, biomarkers helping to detect small HCC would be a great step forward. Recently, the tissue expression of SCCA has been reported in a higher percentage of patients with pre-neoplastic dysplastic lesions than in regenerative nodules. Moreover, in the same study SCCA was reported to be more strongly expressed in premalignant dysplastic nodules than in HCC [9]. In our study a similar statistically significant difference was observed, but because of the small number of patients (5 LC vs. 20 s-HCC patients) with dysplastic cirrhotic nodules, no reliable conclusion can be drawn although it seems an interesting initial observation. Consistently with these data, we report herein that SCCA is more strongly expressed in the tissue of smaller as compared to larger HCC. For the first time, therefore, a marker that can discriminate smaller better than larger nodules is reported, although findings of this marker also in the tissue limit its immediate application to clinical practise. Nevertheless, results in this study contain two different messages, firstly that SCCA could be a biomarker of premalignant transformation and secondly, that the irregular behavior of this serum biomarker could be related to a different biological tumor status.

In particular, the decreased SCCA expression with the progression of tumor size, and the increased expression in the peritumoral tissue of larger HCC at higher risk of further neoplastic transformation support our first conclusion. These results are consistent with our previous observation [5], and are also supported by a previous report by Pontisso et al. showing that in patients with LC progressing to HCC, SCCA was consistently increased [10]. This could explain why SCCA could be unexpectedly increased in some LC patients. In fact, the relation between nodule size and tumoral SCCA in our study suggests that high levels of SCCA expression could anticipate clinical evidence of HCC onset.

On the other hand, the lack of correlation between tissue and serum SCCA levels is disappointing and no explanation is yet forthcoming. However, the fact that SCCA is distributed mainly in the cytosol, not associated to membrane-bound vesicles, and therefore is not properly secreted but rather released in the serum as a consequence of cell lysis, could contribute to clarify this issue [11]. This hypothesis is also confirmed by our previous study showing SCCA expression in some cell lines but not in the conditioned medium, suggesting a defective protein secretion [8].

Moreover, it is noteworthy that the amount of serum SCCA does not depend on its expression level in the peritumoral or LC tissue. The higher ratio in smaller than larger nodules suggests that SCCA is produced and released at different times, likely during the earlier events in HCC progression; this offers another possible explanation of the discrepancy between SCCA serum and tissue levels.

In conclusion, this study suggests that SCCA tissue expression could be a marker for early detection of smaller HCC nodules, and contributes to explain why the data in serum can be somewhat disappointing. In addition, our results suggest that proposed biomarkers need to be carefully investigated and clinically validated in relation to specific biological aspects of HCC, so as to obtain more reliable results.

## Materials and methods

### Tissue and serum collection

Tissue specimens of primary nodules and of the peritumoral area were collected from HCC patients, as well as specimens from LC patients. All the specimens, obtained by surgical biopsy, were fixed in 3.7% formaldehyde and processed for routine histology, while a part of the specimen was immediately snap-frozen in liquid nitrogen and stored at -80°C until use. Serum samples from the same patients were collected before any kind of treatment, and stored at -20°C until use.

Patients were classified as LC and HCC according to EASL criteria, and tumor staging was determined according to the CLIP classification [1,12]; nodule size was determined by concordant US and CT and/or MRI scans. SCCA was quantified in matching sera and tissues of 82 patients, 27 LC and 55 HCC. In the latter patients, tissue expression of SCCA was investigated in both neoplastic and paired peritumoral tissue.

The study was performed in accordance with the Helsinki declaration and informed written consent was obtained from all patients before surgery or liver biopsy and before blood sample collection

### Tissue expression of SCCA

Immunohistochemistry was performed on frozen specimens as previously reported [13]. SCCA was detected using a polyclonal antibody directed against recombinant SCCA purchased from (Hepa-Ab, Xeptagen, Italy) and following the manufacturer's instructions.

The expression of SCCA was measured as μm^2 ^of staining by an appropriate software system (Lucia, Nikon, Corp), already validated in several of our previous reports. Briefly, expression of the SCCA antigen was measured in each section as the total stained area, calculated as the mean of ten randomly chosen microscopic fields. Figures 1 and 2 show the mean and standard deviation among all those calculated. To normalize the quantification of the staining, a negative control (a section incubated without the primary antibody) was included in each experiment, so that the sensitivity of the software was calibrated on the background staining.

### Serum determination of SCCA

Serum determination of the SCCA antigen was carried out using an ELISA kit purchased from Xeptagen (Xeptagen, Naples, Italy) following the manufacturer's instructions as previously reported [8]. Briefly, the SCCA ELISA kit is based on a sandwich system whereby an HRP-conjugate streptavidin secondary antibody is used to reveal the reaction. A standard curve was also included as internal control, and samples were tested in duplicate to ensure reproducible results.

### Statistical analysis

Continuous non Gaussian distributed variables were transformed into natural logarithms and described, after back transformation, as means and 95% confidence intervals. Comparisons between independent groups were performed with Student's t test, and among more than two groups, with a generalized linear model. Multiple comparisons were performed by means of Tukey grouping. Pearson's coefficients were determined to evaluate correlations between continuous variables. To evaluate the prediction of S-SCCA as a function of tissue marker values, a linear regression model was set up with the natural logarithm of S-SCCA as the dependent variable and the natural logarithm of the tumoral and/or peritumoral SCCA value as the independent variable. To evaluate the linear relation of tumoral, peritumoral and serum SCCA as a function of nodule size, a regression model was performed only on single nodule observations.

To evaluate the diagnostic accuracy of serum SCCA a ROC analysis was performed; cut-off value and related sensitivity, specificity, and Youden index were determined.

Qualitative variables were summarized as count and percentage. Comparisons between independent groups were performed with chi-square test or Fisher's exact test when appropriate.

All tests were considered statistically significant at a p-value of < 0.05. Analyses were performed with SAS System software for PC, version 9.1.

## Abbreviations

HCC: Hepatocellular carcinoma; LC: liver cirrhosis; SCCA: squamous cellular carcinoma antigen; AFP: alpha-fetoprotein; -IC: immunocomplex.

## Competing interests

The authors declare that they have no competing interests.

## Authors' contributions

PT performed the statistical analysis. EF, UA, GA, AM and AM performed the experiments. LL supervised the collection of biological samples. SA supervised the project. GG supervised the project and prepared the manuscript. All authors read and approved the manuscript.
